# Assessıng the predıctıve accuracy of blood-based bıomarkers ın neonatal outcomes for pregestatıonal dıabetes mellıtus

**DOI:** 10.61622/rbgo/2025rbgo17

**Published:** 2025-04-30

**Authors:** Ayse Cigdem Bayrak, Erdem Fadiloglu, Haticegul Tuncer, Edip Alptug Kir, Umutcan Kayikci, Ozgur Deren

**Affiliations:** 1 Hacettepe University Faculty of Medicine Department of Obstetrics and Gynecology Division of Maternal Fetal Medicine Ankara Turkey Hacettepe University Faculty of Medicine, Department of Obstetrics and Gynecology, Division of Maternal Fetal Medicine, Ankara, Turkey.

**Keywords:** Pregnancy in diabetes, Pregancy, Infant, newborn, Diabetes *mellitus*, Neuthrophils, Lymphocytes, Monocytes, Respiration, artificial, Maternal age, Gestational age, Biomarkers, Hypoglycemia, Intensive care units, neonatal

## Abstract

**Objective:**

This retrospective study aimed to investigate blood-based immune-inflammatory biomarkers (IIBs) in predicting neonatal outcomes in pregnancies with pregestational diabetes mellitus (PGDM).PIV[(neutrophil×platelet×monocyte)/lymphocyte)], SII (neutrophil×platelet/lymphocyte), and NLR neutrophil/lymphocyte) values were evaluated in all three trimesters, and their correlation with neonatal outcomes was examined.

**Methods:**

We included 82 cases of PGDM pregnancies delivered after 32 weeks. Maternal age, gravidity, parity, types of diabetes, and route of delivery were noted. For neonatal outcomes, we recorded gestational age at birth, birth weight percentile, existence of fetal growth restriction, LGA, neonatal intensive care unit (NICU) requirement, Apgar Score <7 at 1, 5, or 10 minutes, need for positive pressure ventilation (PPV), need for mechanical ventilation, hypoglycaemia, hyperbilirubinemia and the need for phototherapy. PIV, SII and NLR values were calculated in each trimester and their association with adverse neonatal outcomes was analyzed.

**Results:**

We could not detect any consistent and significant correlation between SII and PIV values and adverse neonatal outcomes for each trimester. There was a correlation between 3rd trimester NLR and adverse neonatal outcomes, including APGAR <7, the requirement for PPV and mechanical ventilation (p=0.056, 0.013 and 0.060, respectively).

**Conclusion:**

While SII and PIV values did not consistently correlate with adverse neonatal outcomes throughout each trimester in PGDM pregnancies, 3rd-trimester NLR showed a notable association with the requirement for PPV with statistical significance and with Apgar Score <7 and the requirement for mechanical ventilation without statistical significance. NLR in the third trimester may hold potential as a predictive marker for specific adverse neonatal outcomes in PGDM pregnancies, warranting further investigation.

## Introductıon

Diabetes mellitus (DM) is a group of metabolic diseases characterized by reduced or absent pancreatic insulin secretion, abnormal peripheral insulin sensitivity, or both.^(1)^ During pregnancy, maternal insulin resistance develops under the influence of several factors, especially placenta-derived hormones.^(2)^ Hyperplasia, hypertrophy, and hyperfunction are achieved by the adaptive and dynamic plasticity of beta cells, allowing the pancreas to respond to increased maternal insulin resistance.^(3)^ Due to the impairment of these adaptive mechanisms in patients with pregestational diabetes mellitus (PGDM), insulin resistance can be aggravated throughout pregnancy leading to hyperglycemia. Increased blood glucose levels create a microenvironment that plays a role in the development and progression of diabetes-related complications by triggering the release of pro-inflammatory molecules and increasing free oxygen radicals.^(4)^ Chronic inflammation, especially low-grade chronic inflammation, is associated with insulin resistance and plays a role in the development and progression of diabetes. Also, an impaired balance of immune cells and inflammatory pathways can have a detrimental effect on the developing fetus.^(5)^ Epidemiological studies have shown that an unfavourable intrauterine environment during pregnancy can be the first step in the development of diseases and adverse perinatal outcomes.^(6)^

In recent years, some blood-based immune-inflammatory biomarkers (IIBs) have been used to predict prognosis in various diseases, especially in the oncological field. In 2014, the Systemic Immune Inflammation Index (SII) (neutrophil×platelet/lymphocyte) was defined for the practical estimation of inflammation in patients with hepatocellular carcinoma.^(7)^ Subsequently, this relationship has since been investigated in other diseases exacerbated with inflammation. Over the last few years, several studies have been conducted to correlate SII with adverse perinatal outcomes in specific obstetric complications.^(8,9)^

Another index used as an indicator of inflammation is the Pan-Immune Inflammation Value (PIV). Studies indicate that changes in PIV may help in disease monitoring and prognostic assessment in various inflammatory disorders. The PIV has been reported to outperform other well-known and widely investigated immune biomarkers such as NLR and SII in predicting prognosis in various diseases.^(10,11)^ However, according to our literature research, PIV has never been studied in pregnant women for predicting any adverse perinatal outcome.

Neutrophil-to-lymphocyte ratio (NLR) (neutrophil/lymphocyte) is among the most widely used IIBs in the recent years. NLR has been studied in almost every subspeciality, including obstetrics and pregnant women.^(12,13)^

The possible association between inflammatory markers and adverse neonatal outcomes in patients with PGDM has not been questioned in previous studies. It is well known that PGDM is associated with many adverse perinatal outcomes, and altered blood glucose levels are associated with adverse outcomes. Thus, we hypothesized that IIBs may be correlated with adverse outcomes in pregnancies with PGDM. In this study, we aimed to investigate the effect of PIV, SII and NLR in predicting neonatal outcomes in patients with PGDM.

## Methods

The study population was obtained from the electronic medical records of pregnant women with PGDM who were followed up and delivered at the Division of Perinatal Medicine, Department of Obstetrics and Gynecology, Hacettepe University. We searched all delivery records between January 2016 and April 2023. We included patients with PGDM who had a complete blood count in all 3 trimesters and delivered in our institution. We excluded patients with incomplete or missing data, multiple gestations, or fetuses diagnosed with major congenital anomalies or chromosomal abnormalities. Furthermore, we excluded cases that resulted in stillbirth, including those involving the termination of pregnancy due to fetal or maternal indications. To minimize potential biases and confounding factors, we also excluded preterm deliveries occurring before the 32nd gestational week to avoid the disproportionate influence of extreme prematurity on our outcomes.

Maternal age, gravidity, parity, type of PGDM, and route of delivery were noted. For neonatal outcomes, we recorded gestational age at birth (week), birth percentile, fetal growth restriction (birth weight percentile < 10p), large for gestational age (LGA) (birth weight percentile > 90p), neonatal intensive care unit (NICU) requirement, Apgar Score <7 (any Apgar score < 7 at the first 10 minutes), need for positive pressure ventilation (PPV), need for mechanical ventilation, hypoglycaemia (recurrent blood glucose level of <2.6 mmol/L following normal feeding within a 12-hour period), hyperbilirubinaemia (based on gestational age and hour of life), need for phototherapy, and the detection of at least one of these was defined as a composite adverse outcome.

The PIV, SII, and NLR values were calculated by recording hemoglobin, leucocyte, neutrophil, lymphocyte, monocyte, and platelet values in each trimester. PIV, SII and NLR were calculated as follows: [(neutrophil × platelet × monocyte) / lymphocyte], (neutrophil × platelet / lymphocyte) and (neutrophil/lymphocyte).

We performed visual and quantitative analyses for the determination of the normality of distribution. Due to a lack of normal distribution, the quantitative variables were given as median values and minimum-maximum. We performed the Mann-Whitney test for independent continuous variables. Friedman test was used for the comparison of non-parametric dependent variables. All statistical calculations were performed using the Statistical Package for Social Sciences (SPSS) for Windows (SPSS version 23) software package. A p value < 0.05 was regarded as statistically significant.

Formal ethical approval was not necessary due to the retrospective design of the study.

## Results

This retrospective study included a total of 82 pregnancies with PGDM. Among these, there were 36 cases of Type 1 DM and 46 cases of Type 2 DM. Our initial analysis was performed to assess maternal age, gravidity, parity, route of delivery, birth weight, gestational age at birth, and the rate of fetal complications (Table 1). Out of a total of 82 deliveries, 31 (37.8%) were preterm deliveries, with only five cases occurring between the 32nd and 34th gestational weeks. Table 2 displays hemoglobin, neutrophil, lymphocyte, monocyte, platelet values and the PIV, SII and NLR in all three trimesters. We detected an increasing trend in NLR and PIV throughout the pregnancy, while SII was highest in the 2nd trimester. Additionally, all complete blood count parameters were found to be significantly different among trimesters except leucocyte count (p<0.05 for all).

**Table 1:** Demographic Information of the patients and rates of complications

Variables		n(%)
Maternal age*		31.5 (19 – 46)
Gravide*		2 (1 – 9)
Parity*		1 (0 – 6)
Diagnosis of diabetes mellitus**		
	Type 1 DM	36(43.9)
	Type 2 DM	46(56.1)
Route of delivery**		
	Vaginal delivery	8(9.8)
	Cesarean section	74(90.2)
Birth weight*		3175(1700 – 4910)
Birth week*		37(32 – 41)
	Complications**	
	NICU admission	37(45.1)
	FGR	11(13.4)
	LGA	32(39)
	APGAR < 7	12(14.6)
	PPV	16 (19.5)
	Mechanic ventilation	17(20.7)
	Hypoglycemia	16(19.5)
	Hyperbilirubinemia	18(22)
	Phototherapy	15 (18.3)
	Composite adverse outcome	45 (54.8)

* Median and minimum-maximum values are presented;

** Number and rate of event are presented

**Table 2:** Distribution of complete blood count parameters and inflammatory markers according to trimesters

	1st trimester	2nd trimester	3rd trimester	p-value
Hb (g/dL)	12.9 (10.5 – 15.7)	12.2 (8.8 – 14.5)	11.7 (8.3 – 16.0)	<0.001
Leukocyte (10^3^/μL)	9.4 (3.3 – 15.5)	9.7 (5.8 – 18.3)	10.0 (4.2 – 20.2)	0.233
Neutrophil (10^3^/μL)	6.3 (2.4 – 12.1)	7.2 (3.5 – 16.7)	7.3 (2.0 – 17.6)	0.047
Lymphocyte (10^3^/μL)	2.12 (0.69 – 3.90)	1.80 (1.10 – 4.20)	1.80 (0.90 – 3.20)	0.001
Monocyte (10^3^/μL)	0.53 (0.16 – 1.10)	0.60 (0.27 -1.20)	0.60 (0.27 – 1.20)	0.050
Platelet (10^3^/μL)	243 (176- 446)	225 (145 - 395)	214 (99 – 460)	0.002
NLR	3.07 (1.37 – 5.94)	3.72 (1.58 – 13.05)	3.89 (1.20 – 14.22)	<0.001
SII	704 (266 – 1728)	857 (280 – 2818)	843 (240 – 2417)	0.017
PIV	399 (111 – 1210)	488 (84 – 1948)	547 (69 – 1692)	0.009

Friedman test was used for comparison of dependent variables

Analyses were performed to investigate a possible association between adverse neonatal outcomes and IIBs during each trimester of pregnancy. We examined the correlation between the indices and the neonatal outcomes as defined in the methods section.

The analysis of the correlation between SII and adverse neonatal outcomes across all three trimesters did not reveal any clinically significant finding. Furthermore, the third trimester SII value was statistically lower in neonates admitted to the NICU, contrary to the expected association with adverse neonatal outcomes. Hypoglycemia was also statistically significant in the second trimester in the group with high SII, but this relationship could not be confirmed in the first and third trimesters (Table 3).

**Table 3:** Associaton of SII values and adverse neonatal outcomes in each trimester

	SII 1st Trimester		SII 2nd Trimester		SII 3rd Trimester	
	Yes	No	Yes	No	Yes	No
NICU	690 (266 – 1728) p: 0.608	886 (334 – 1548)	823 (280 – 2818) p: 0.540	886 (334 – 1548)	654 (240 – 2266) p: 0.014	925 (325 – 2417)
FGR	681 (570 – 1267) p: 0.668	715 (266 – 1728)	888 (433 – 2818) p: 0.695	835 (280 – 2783)	864 (357 – 1440) p: 0.966	829 (240 – 2417)
LGA	731 (266 – 1353) p: 0.794	704 (352 – 1728)	831 (280 – 1534) p: 0.877	865 (428 – 2818)	709 (325 – 1575) p: 0.278	906 (240 – 2417)
APGAR < 7	661 (367 – 1351) p: 0.668	724 (266 – 1728)	865 (408 – 1464) p: 0.963	846 (280 – 2818)	929 (531 – 2266) p: 0.738	848 (240 – 2417)
PPV	690 (266 – 1351) p: 1.000	735 (352 – 1728)	861 (428 – 2818) p: 0.811	835 (280 – 2783)	815 (240 – 1672) p: 0.958	853 (325 – 2417)
Mechanic Ventilation	688 (266 – 1351) p: 0.761	739 (352 – 1728)	835 (428 – 2818) p: 0.603	871 (280 – 2783)	815 (240 – 2266) p: 0.958	853 (325 – 2417)
Hypoglycemia	786 (266 – 1728) p: 1.000	695 (352 – 1645)	1188 (280 – 2818) p: 0.043	815 (334 – 1593)	715 (344 – 2000) p: 0.577	866 (240 – 2417)
Hyperbilirubinemia	596 (266 – 1077) p: 0.761	739 (352 – 1728)	819 (408 – 1593) p: 0.453	876 (280 – 2818)	654 (357 – 2266) p: 0.056	906 (240 – 2417)
Phototherapy	671 (367 – 1353) p: 0.786	751 (266 – 1728)	882 (408 – 1593) p: 0.733	819 (280 – 2818)	778 (357 – 1351) p: 0.658	876 (240 – 2417)
Composite Adverse Outcome	724 (266 – 1728) p: 0.799	689 (352 – 1645)	886 (280 – 2818) p: 0.111	780 (334 – 1548)	787 (240 – 2266) p: 0.732	876 (325 – 2417)

In all three trimesters, no correlation was observed between PIV and other neonatal outcomes (Table 4). Additionally, PIV was found to be lower in neonates admitted to the NICU during the third trimester, similar to the finding with SII.

**Table 4:** Associaton of PIV values and adverse neonatal outcomes in each trimester

	PIV 1st Trimester		PIV 2nd Trimester		PIV 3rd Trimester	
	Yes	No	Yes	No	Yes	No
NICU	347 (111 – 1210) p: 0.608	406 (116 – 947)	481 (84 – 1948) p: 0.897	527 (198 – 1545)	380 (69 – 1621) p: 0.014	619 (88 – 1692)
FGR	373 (259 – 633) p: 0.668	399 (111 – 1210)	554 (208 – 1274) p: 0.695	475 (84 – 1948)	623 (178 – 973) p: 0.966	544 (69 – 1692)
LGA	349 (133 – 947) p: 0.794	410 (111 – 1210)	467 (84 – 1545) p: 0.251	534 (171 – 1948)	460 (88 – 945) p: 0.278	590 (69 – 1692)
APGAR < 7	291 (197 – 946) p: 0.198	410 (111 – 1210)	555 (200 – 1317) p: 0.963	487 (84 – 1948)	616 (266 – 1586) p: 0.738	541 (69 – 1692)
PPV	398 (133 – 946) p: 1.000	400 (111 – 1210)	483 (171 – 1317) p: 0.811	488 (84 – 1948)	723 (69 – 1621) p: 0.958	529 (88 – 1692)
Mechanic ventilation	372 (133 – 946) p: 0.761	403 (111 – 1210)	491 (171 – 1274) p: 0.949	487 (84 – 1948)	723 (69 – 1621) p: 0.958	529 (88 – 1692)
Hypoglycemia	323 (116 – 1210) p: 1.000	400 (111 – 969)	713 (84 – 1948) p: 0.171	471 (198 – 1545)	453 (137 – 1400) p: 0.958	549 (69 – 1692)
Hyperbilirubinemia	306 (133 – 969) p: 0.362	410 (111 – 1210)	509 (171 – 1545) p: 0.839	488 (84 – 1948)	411 (178 – 1621) p: 0.177	555 (69 – 1692)
Phototherapy	346 (116 – 969) p: 0.415	410 (111 – 1210)	564 (171 – 1545) p: 0.397	475 (84 – 1948)	551 (178 – 1621) p: 0.943	544 (69 – 1692)
Composite adverse outcome	406 (116 – 1210) p: 0.799	383 (111 – 836)	574 (84 – 1948) p: 0.111	468 (198 – 1199)	554 (69 – 1621) p: 0.560	498 (88 – 1692)

A statistically significant correlation was identified between the requirement for PPV and NLR. However, while no direct correlation emerged with other adverse outcomes, a noteworthy proximity to a p-value of 0.05 was observed concerning the requirement for mechanical ventilation and an Apgar score below 7 (p: 0.060 and 0.053, respectively) (Table 5).

**Table 5:** Associaton of NLR values and adverse neonatal outcomes in each trimester

	NLR 1st Trimester		NLR 2nd Trimester		NLR 3rd Trimester	
	Yes	No	Yes	No	Yes	No
NICU	3.15 (1.44 – 5.71) p: 0.608	2.85 (1.37 – 5.94)	3.80 (1.67 – 13.05) p: 0.897	3.69 (1.58 – 6.00)	4.13 (1.20 – 11.75) p: 0.205	3.77 (1.38 – 14.22)
FGR	2.75 (2.33 – 4.63) p: 0.668	3.11 (1.37 – 5.94)	4.28 (1.82 – 13.05) p: 0.695	3.66 (1.58 – 12.54)	3.89 (2.17 – 7.92) p: 0.966	3.88 (1.20 – 14.22)
LGA	2.78 (1.44 – 5.23) p: 0.192	3.12 (1.37 – 5.94)	3.79 (1.58 – 6.21) p: 0.877	3.72 (1.82 – 13.05)	3.72 (1.75 – 5.93) p: 0.540	4.21 (1.20 – 14.22)
APGAR < 7	2.83 (1.60 – 5.19) p: 0.668	3.11 (1.37 – 5.94)	3.14 (2.31 – 5.90) p: 0.963	3.80 (1.58 – 13.05)	4.77 (2.83 – 7.93) p: 0.056	3.77 (1.20 – 14.22)
PPV	3.11 (1.47 – 5.19) p: 1.000	2.91 (1.37 – 5.94)	4.58 (2.50 – 13.05) p: 0.338	3.68 (1.58 – 12.54)	5.18 (1.20 – 11.75) p: 0.013	3.65 (1.38 – 14.22)
Mechanic ventilation	3.07 (1.47 – 5.19) p: 0.761	3.05 (1.37 – 5.94)	3.66 (2.50 – 13.05) p: 0.949	3.81 (1.58 – 12.54)	4.76 (1.20 – 11.75) p: 0.060	3.72 (1.38 – 14.22)
Hypoglycemia	3.15 (1.47 – 5.71) p: 0.506	3.00 (1.37 – 5.94)	5.10 (1.67 – 13.05) p: 0.171	3.67 (1.58 – 6.00)	4.21 (2.05 – 9.26) p: 0.958	3.87 (1.20 – 14.22)
Hyperbilirubinemia	2.62 (1.44 – 5.19) p: 0.362	3.11 (1.37 – 5.94)	3.67 (2.10 – 5.86) p: 0.839	3.88 (1.58 – 13.05)	4.20 (2.26 – 11.75) p: 0.365	3.84 (1.20 – 14.22)
Phototherapy	2.84 (1.44 – 5.19) p: 0.787	3.11 (1.37 – 5.94)	4.04 (2.10 – 5.86) p: 0.397	3.63 (1.58 - 13.05)	3.90 (2.26 – 11.75) p: 0.943	3.81 (1.20 – 14.22)
Composite adverse outcome	3.11 (1.44 – 5.71) p: 0.444	2.85 (1.37 – 5.94)	4.04 (1.67 – 13.05) p: 0.111	3.46 (1.58 – 6.00)	4.05 (1.20 – 11.75) p: 0.296	3.55 (1.38 – 14.22)

Further analyses were conducted to compare inflammatory indices between patients who delivered at term and those who delivered preterm (Table 6). No statistically significant differences were observed between the two groups in any of the inflammatory indices measured across all trimesters. The causes of preterm delivery in this cohort were categorized as follows: preterm labor (48.3%), fetal distress (29.1%), preeclampsia (16.1%), and others (6.45%). These were also analyzed in relation to inflammatory indices, with no significant associations found.

**Table 6:** Comparison of ınflammatory ındices between patients with and without preterm delivery

	Preterm delivery	Term delivery	p-value
SII 1st trimester	714 (266 – 1728)	695 (352 – 1645)	1.000
SII 2nd trimester	856 (334 – 2818)	857 (280 – 1548)	0.884
SII 3rd trimester	704 (240 – 2266)	888 (325 – 2417)	0.269
PIV 1st trimester	414 (133 – 1210)	350 (111 – 947)	0.599
PIV 2nd trimester	546 (208 – 1948)	471 (84 – 1545)	0.185
PIV 3rd trimester	515 (69 – 1586)	551 (88 – 1692)	0.881
NLR 1st trimester	3.12 (1.47 – 5.71)	3.00 (1.37 – 5.94)	0.599
NLR 2nd trimester	3.78 (1.58 – 13.05)	3.72 (1.67 – 6.00)	0.884
NLR 3rd trimester	4.02 (1.20 – 7.93)	3.85 (1.38 – 14.22)	0.743

## Dıscussıon

There is growing interest in utilizing blood-based IIBs, such as the SII, PIV, and NLR, to predict outcomes in various medical conditions. Initially explored in oncology, these indices have since expanded to inflammatory and obstetric diseases, reflecting the complex interplay between immunity and inflammation. As composite markers, these indices integrate key blood components, notably neutrophils, lymphocytes, and platelets. Although each index is calculated differently, they share common trends due to their reliance on overlapping parameters.

Pregnancy involves significant immune adaptations, shifting from Th2 dominance in early gestation to a pro-inflammatory Th1 state in late gestation.^(14)^ While these adaptations are crucial for pregnancy maintenance and parturition, excessive immune activation can result in obstetric complications like preeclampsia, gestational diabetes mellitus(GDM), or preterm labor.^(15)^ Elevated inflammatory mediators in pregnancies complicated by diabetes may influence adverse neonatal outcomes.^(16)^ Studies have investigated the relationship between inflammation and pregnancy outcomes in patients with PGDM using different markers, but there has not been a significant focus on IIBs.^(17)^

In this study, we hypothesized that trimester-specific variations in IIBs might correlate with neonatal outcomes in PGDM pregnancies. We observed a progressive increase in all three indices from the first to the third trimester, largely driven by rising neutrophil counts and a reduction in lymphocyte and platelet counts. These trends seem to reflect physiological gestational changes, suggesting that such increases may represent adaptive processes rather than pathological states. However, exceeding certain thresholds may signal adverse outcomes, warranting their potential use as predictive tools. Beyond placental permeability, increased inflammation may extend beyond the maternal compartment and potentially influence fetal immune programming through shared inflammatory mediators.^(18)^

Individually, SII was significantly associated with neonatal hypoglycemia in the second trimester, but it did not correlate with other adverse outcomes. Similarly, PIV showed inconsistent relationships with composite neonatal outcomes. Interestingly, lower SII and PIV levels in infants requiring NICU admission in the third trimester contradicted expectations. NLR, like other IIBs, increased toward the third trimester and showed a stronger association with adverse neonatal outcomes. In the 3rd trimester, NLR values were significantly elevated in infants requiring PPV at birth. Moreover, among composite neonatal outcomes, a higher NLR was observed in infants who required mechanical ventilation or had an Apgar score <7, although statistical significance was narrowly missed (p-value close to 0.05). This finding may be the most notable in our study, suggesting that 3rd-trimester NLR may be a more reliable prognostic marker for neonatal respiratory compromise.

In patients with diabetes, elevated IIBs have been associated with microvascular complications, poor glycemic control, and increased inflammatory status, which in turn exacerbate diabetes-related complications like diabetic nephropathy and retinopathy.^(19,20)^ Similarly, in obstetric populations, elevated inflammatory markers have been linked to poor neonatal outcomes in GDM pregnancies, such as low birth weight and higher NICU admission rates.^(16)^ Our study, the first to explore these indices in PGDM, showed that contrary to expectations, lower SII and PIV levels were associated with NICU admission, a finding that warrants further investigation. This paradox may be attributed to confounding factors such as gestational age or fetal growth restriction, which were not fully accounted for in this analysis. Alternatively, the unique metabolic changes in PGDM pregnancies might alter the influence of these indices compared to other obstetric populations, emphasizing the need for context-specific evaluations.

Pro-inflammatory cytokines produced during maternal microinflammation can cross the placenta, influencing fetal immune programming. While this may help prepare the fetus for postnatal immune challenges, it could also predispose the offspring to metabolic or neurodevelopmental disorders later in life.^(21,22)^ Excessive maternal inflammation may disrupt placental function, potentially leading to fetal growth restriction or other complications.^(23,24)^ Recent animal studies suggest that maternal immune activation can induce altered inflammatory reactivity in offspring, which may result in either protective adaptations or maladaptive immune responses.^(21)^ These findings point to the need for further research to explore how maternal systemic inflammation impacts fetal development and its long-term effects.

Our analysis also revealed that preterm delivery does not have a significant correlation with the inflammatory indices measured in this study. This aligns with our primary findings and adds robustness to the data by addressing a potential source of bias. While some previous studies have reported higher levels of inflammatory biomarkers in association with preterm delivery,^(25)^ our cohort did not demonstrate such a relationship. This may reflect differences in study populations, methodologies, or the specific inflammatory indices analyzed. However, we acknowledge that preterm delivery remains a complex condition influenced by multiple factors, and it is possible that other unmeasured variables associated with preterm birth could still introduce residual confounding.

Limitations of our study include its retrospective nature, the relatively small sample size, and the predominance of cesarean deliveries in our cohort, which restricts the generalizability of our findings. Additionally, while our analyses showed no significant differences in inflammatory indices between preterm and term deliveries, we acknowledge that residual confounding factors related to preterm birth may still exist, potentially impacting the applicability of our results to broader populations.

## Conclusion

Despite these limitations, this is the first study to investigate the relationship between IIBs and neonatal outcomes in PGDM pregnancies. To enhance the reliability of our findings, we employed strict inclusion and exclusion criteria to minimize confounders and ensure a focused analysis.
